# Causal Effects of Oxidative Stress on Diabetes Mellitus and Microvascular Complications: Insights Integrating Genome-Wide Mendelian Randomization, DNA Methylation, and Proteome

**DOI:** 10.3390/antiox13080903

**Published:** 2024-07-26

**Authors:** Kang Liu, Zitong Chen, Lishan Liu, Ting Li, Changying Xing, Feng Han, Huijuan Mao

**Affiliations:** 1 Department of Nephrology, The First Affiliated Hospital of Nanjing Medical University (Jiangsu Province Hospital), Nanjing 210029, China; 2 International Joint Laboratory for Drug Target of Critical Illnesses, Key Laboratory of Cardiovascular and Cerebrovascular Medicine, School of Pharmacy, Nanjing Medical University, Nanjing 211166, China; 3 Institute of Brain Science, The Affiliated Brain Hospital of Nanjing Medical University, Nanjing 211166, China

**Keywords:** oxidative stress, diabetes mellitus, microvascular complications, summary data-based Mendelian randomization

## Abstract

Background: Oxidative stress (OS) is involved in the development of diabetes, but the genetic mechanisms are not completely understood. We integrated multi-omics data in order to explore the genetic relations between OS-related genes, diabetes mellitus, and microvascular complications using Mendelian randomization and colocalization analysis. Methods: Summary-level data related to OS were acquired from respective studies of methylation, expression, and protein abundance quantitative trait loci. Genetic associations concerning diabetes, diabetic nephropathy (DN), and diabetic retinopathy (DR) were derived from the FinnGen study. Summary-data-based Mendelian randomization (SMR) analysis was conducted to evaluate the correlations between molecular features concerned with OS-related genes and diabetes mellitus, along with its microvascular complications. Additionally, we performed colocalization analysis to determine if the detected signal pairs shared a causal genetic variant. Results: At the genetic level, we identified ten potential causal associations of oxidative stress genes with diabetes, along with microvascular complications, through SMR and colocalization analysis. After integrating the DNA methylation quantitative trait loci (mQTL) and expression QTL (eQTL) data, our analyses revealed a correlation between the methylation site cg26343298 and reduced expression of TP53INP1, supporting the protective role of cg26343298 methylation on type 2 diabetes (T2D) and diabetic nephropathy. Similarly, an inverse association was observed between gene methylation and expression in CHEK1 (cg07110182), confirming the beneficial effect of modification of CHEK1 by cg07110182 in diabetic retinopathy. In addition, upregulation of SUOX expression by cg22580629 was linked to a reduced risk of diabetic retinopathy. At circulating protein levels, genetically predicted a higher level of ICAM1 (OR 1.05, 95%CI 1.03–1.08) was positively connected with the risk of diabetic retinopathy. Conclusions: This SMR study elucidated that the TP53INP1 gene was putatively associated with T2D and DN risk, while the SUOX and CHEK1 genes were associated with DR risk through oxidative stress mechanisms. Additionally, our study showed a positive correlation between the ICAM-1 protein and DR. These findings may enhance our understanding of their pathogenesis and suggest new therapeutic targets for clinical practice.

## 1. Introduction

Diabetes mellitus (DM) is a chronic disease affecting approximately 6.1% of the global population, and its prevalence continues to rise [1]. Diabetic nephropathy (DN) and diabetic retinopathy (DR) are the most common and serious complications, significantly reducing the quality of life for DM patients [2]. The pathogenesis of DM remains largely unknown, while genetic predisposition, an unhealthy diet, and lifestyle play important roles in the current epidemic [3]. Addressing diabetes and its complications remains a persistent societal challenge.

Oxidative stress (OS), defined by an imbalance between oxidants and antioxidants, compromises redox signaling and regulation, resulting in molecular damage [4]. High glucose-induced generation of OS is known to lead to resistance to insulin and impaired β-cell function, contributing to the pathogenesis and progression of diabetes mellitus [5]. Genome-wide association studies (GWAS) have recently been utilized to locate genetic loci that include OS genes linked to diabetes [6,7]. Nevertheless, due to the intricate linkage disequilibrium (LD) patterns within the genome, top-associated variants may not be causally related [8]. The precise OS-related genes and their subsequent impact on diabetes and its complications are still not clearly understood.

DNA methylation (DNAm) levels have been found to be closely associated with the incidence of diabetes [9]. Abnormal epigenetic modifications in cells are closely associated with oxidative stress. The transcriptional levels of genes associated with redox balance are regulated by the methylation status of the cytosine in the DNA molecule. The methylation of CpG can change the accessibility of functional factors to the promoter or enhancer regions, bringing interference to the protein–DNA interaction [10]. Previous studies have suggested that diabetes-related cytokines regulate epigenetics by altering DNA methylation in islet β cells, leading to changes in gene expression of inflammatory and immune pathways [11]. Notably, the OS-related variations are likely to regulate DNAm, gene expression, and protein levels [12], which will provide new ideas for finding therapeutic targets.

As a modern epidemiological method, two-sample mendelian randomization (MR) employs genetic variants as instrumental variables to determine possible causal links between exposure and outcome variables. Summary-data-based Mendelian randomization (SMR) extends this conception and is mainly used to analyze relationships among genotype, gene expression, and phenotype. Here, with the application of SMR, we integrated large-scale GWAS statistics with expression quantitative trait loci (eQTL), DNA methylation QTL (mQTL), and protein QTL (pQTL) in the blood and pancreas to explore potential associations between gene expression, DNAm, and protein expression of OS-related genes and the risks of diabetes mellitus, diabetic nephropathy, and diabetic retinopathy.

## 2. Methods

### 2.1. Study Design

**Figure 1:**
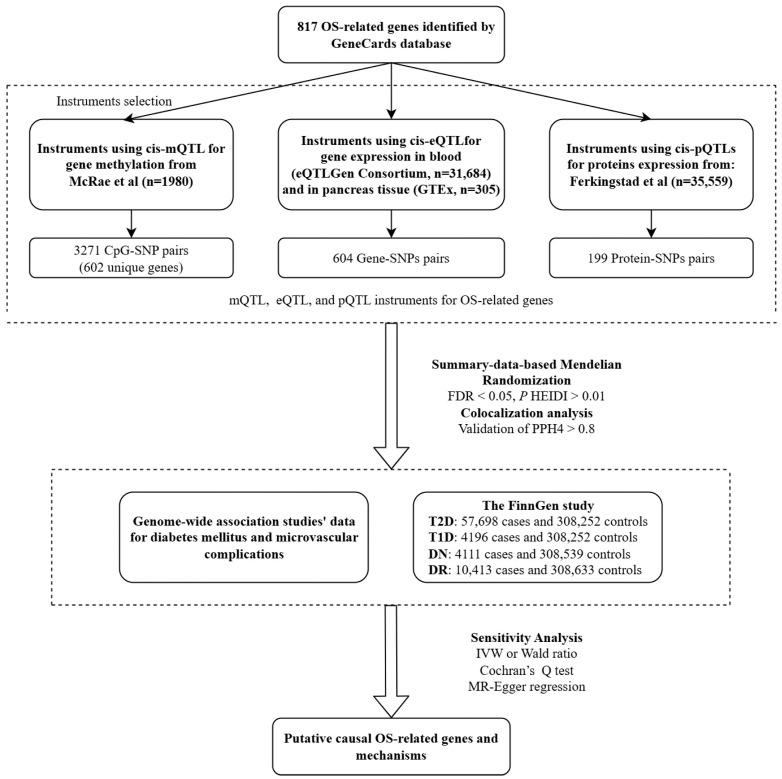
Summary of the study design and workflow. OS, oxidative stress; QTL, quantitative trait loci; SNP, single nucleotide polymorphisms; FDR, false discovery rate; HEIDI, Heterogeneity in Dependent Instrument test; PPH4, posterior probability of H4; T2D, type 2 diabetes; T1D, type 1 diabetes; DN, diabetic nephropathy; DR, diabetic retinopathy; IVW, inverse variance weighted.

The total design scheme of this study is shown in Figure 1. The quantitative trait loci and genome-wide association studies providing summary-level data are openly accessible (Table S1). The studies received approval from their respective institutional review boards.

### 2.2. Exposure Sources

Genes were retrieved and acquired in the GeneCards database (v5.10) using the keyword “oxidative stress” and filtered based on a relevance score of ≥7, following established methodologies [13,14,15]. A total of 817 OS-related genes were identified (Table S2). The quantitative trait loci (QTL), genetic variants influencing molecular traits [16], demonstrate the relationship between single nucleotide polymorphisms (SNPs) and various biological markers, containing DNA methylation, gene expression, and protein expression levels. Associations between SNPs and CpG sites were analyzed using blood DNA methylation QTL (mQTL) data on 1980 individuals of European ancestry reported by McRae et al. [17]. Only the DNA methylation probes with at least a cis-mQTL at *p* < 5 × 10^−8^ and only SNPs within 2 Mb distance from each probe are available. Blood expression quantitative trait loci (eQTL) data were sourced from the eQTLGen consortium, which encompasses 31,684 individuals [18]. Every SNP-gene combination with a distance <1 Mb and tested in at least 2 cohorts was included. Additionally, tissue-specific eQTL data were obtained from the Genotype-Tissue Expression (GTEx) project using the GTEx v8 dataset, which includes 52 tissue samples and two cell lines across 838 donors [19]. Our focus was on eQTL data from pancreatic tissue. The BESD format for mQTL and eQTL data were obtained from the SMR website and the eQTLGen organization. For proteins, we extracted summary statistics of genetic associations from information provided by Ferkingstad et al., which focused on protein quantitative trait loci (pQTL) and included 35,559 individuals from Iceland [20]. The pQTLs were generated from genome-wide association testing using the 4907 aptamer levels adjusted for rank-inverse normal transformed for age, sex, and sample age as phenotypes and 27.2 million imputed variants as genotypes. Then, the pQTL summary data were transformed to BESD format according to standard procedures on the SMR website. After screening for OS-related genes, we identified 602 methylation sites, 604 expressed genes, and 199 proteins using the mQTL, eQTL, and pQTL datasets, respectively, with significance thresholds of *p* < 5 × 10^−8^.

### 2.3. Outcome Sources

Genetic associations with diabetes mellitus and its microvascular complications were sourced from the R9 data release of the FinnGen study, which is publicly accessible [21]. The diagnosis of diabetes mellitus and its microvascular complications was based on ICD codes, comprising 57,698 cases for type 2 diabetes with 308,252 controls, 4196 cases for type 1 diabetes with 308,252 controls, 4111 cases for diabetic nephropathy with 308,539 controls, and 10,413 cases for diabetic retinopathy with 308,633 controls (Table S1). The population of the study was limited to individuals of European ancestry.

### 2.4. Statistical Analyses

#### 2.4.1. Summary-Data-Based MR Analysis

Summary-data-based Mendelian randomization (SMR) was utilized to assess the relationship of methylation, expression, and protein abundance of genes related to OS with the risks of diabetes mellitus (DM) and its associated microvascular issues. By leveraging top-associated cis-QTL, SMR achieves significantly greater statistical power compared to traditional Mendelian randomization analyses, particularly for exposure and consequence data derived from two separate large cohorts [12]. Focusing on SNPs within a ±1000 kb window surrounding each gene, primary cis-QTLs were identified, with a *p*-value threshold of <5 × 10^−8^. The LD estimation was performed by using genomes of European ancestry obtained from the 1000 Genomes Project Consortium as references [22]. SNPs showing allele frequency differences greater than 0.2 between pairwise datasets (including the GWAS summary data, the mQTL summary data, the eQTL summary data, the pQTL summary data, and the LD reference data) were excluded. The causal association was calculated as follows: β_SMR_ = β_SNP-GWAS_/β_SNP-QTL_, β_SMR_ is calculated as the estimated effect size of oxidative stress (OS)-related genes on diabetes or complications GWAS, where β_SNP-QTL_ is the estimated effect size of SNP on OS-related genes (a genetic variant–exposure trait association) and β_SNP-GWAS_ is the estimated effect size of SNP on diabetes or complications (the same genetic variant–outcome trait association). β > 0 indicates a positive association, while β < 0 indicates a negative association. Odds ratios (ORs) for the impact of OS-related genes on DM and its microvascular complications quantify the effect of a quantitative trait locus, which was calculated as follows: OR = exp (β_SMR_), where exp represents the base of the natural logarithm. To differentiate pleiotropy from linkage, the Heterogeneity in Dependent Instrument (HEIDI) test was applied, with a P-HEIDI < 0.01 indicating probable pleiotropy, leading to exclusion from further analysis. These analyses were conducted using the SMR software, Win version 1.3.1, available at (https://yanglab.westlake.edu.cn/software/smr/#Overview, accessed on 23 March 2024). Then, the reference genotype data, GWAS summary statistics data, and BESD-formatted QTL summary data were used as SMR inputs. Other settings were set as the default arguments. To control the rate of type I error, we adjusted the P_SMR_ value with the Benjamini–Hochberg method to account for multiple testing [23]. A false discovery rate (FDR)-adjusted *p*-value < 0.05 was defined as statistical significance. Only associations with an FDR-adjusted *p*-value < 0.05 and P-HEIDI > 0.01 were selected to conduct colocalization analysis.

#### 2.4.2. Colocalization Analysis

Colocalization analysis, a method that enhances genetic studies by identifying shared genetic variants associated with both exposure and outcome, reports five posterior probabilities corresponding to distinct hypotheses. These hypotheses include the following: (H0) no causal variants for either trait; (H1) a causal variant only affecting gene expression; (H2) a causal variant only influencing disease risk; (H3) different causal variants for each trait; and (H4) a single variant affecting both traits. For analyses such as pQTL-, eQTL-, and mQTL-GWAS, the regions investigated extended ±1000 kb, ±1000 kb, and ±500 kb from the locus separately. The analysis, typically executed using the “coloc” package in R, utilizes default parameters (p1 = 1 × 10^−4^, p2 = 1 × 10^−4^, and p12 = 1 × 10^−5^) and considers a posterior probability higher than 0.80 for hypothesis H4 as indicative of shared genetic effects between the traits.

#### 2.4.3. Sensitivity Analysis

To test for the significant associations found in the primary SMR analyses, sensitivity analyses were conducted with three additional MR methods, including inverse variance weighted, MR Egger, and weighted median, by using the “TwoSampleMR” R package. F-statistic was used to calculate the strength of the variants, and a SNP with a value >10 was considered a strong MR instrument [24]. We tested for heterogeneity across the individual causal effects using the Cochran’s Q statistic implemented in both the MR Egger and IVW methods, where the *p*-value of Cochran’s Q test < 0.05 indicates the existence of heterogeneity [25]. Considering that pleiotropy could introduce bias into MR results, we employed MR Egger regression to explore its potential influence. There are no directional pleiotropies if an intercept is close to zero, where the *p*-value is >0.05 [26].

## 3. Results

### 3.1. SMR Analysis of Cis-eQTL and Diabetes Outcomes

**Figure 2:**
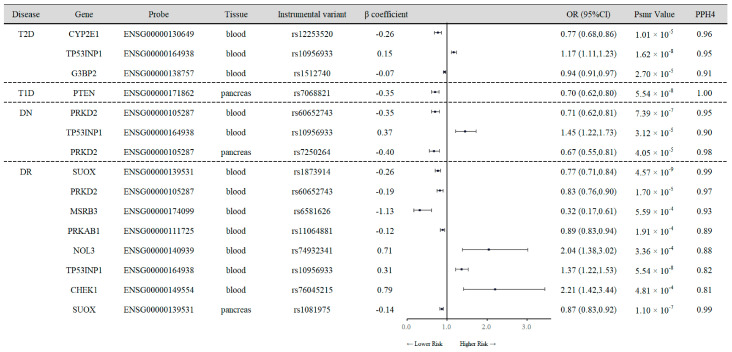
Mendelian randomization results for the association between the expression of OS-related genes and the risk of diabetes mellitus and microvascular complications. T2D, type 2 diabetes; T1D, type 1 diabetes; DN, diabetic nephropathy; DR, diabetic retinopathy; OR, odds ratio; CI, confidence interval; PPH4, posterior probability of H4. CYP2E1−cytochrome P450 family 2 subfamily E member 1; TP53INP1−tumor protein P53-inducible nuclear protein 1; G3BP2−G3BP stress granule assembly factor 2; PTEN−phosphatase and tensin homolog; PRKD2−protein kinase D2; SUOX−sulfite oxidase; MSRB3−methionine sulfoxide reductase B3; PRKAB1−protein kinase AMP-activated non-catalytic subunit beta 1; NOL3−nucleolar protein 3; CHEK1−checkpoint kinase 1.

To control for genome-wide type I errors, FDR corrections were applied with a significance threshold set at *p* < 0.05. Subsequent application of the HEIDI test, with a threshold of P-HEIDI > 0.01, allowed us to identify 44 association signals at 37 unique genetic loci in blood and 7 association signals at 6 unique genetic loci in pancreatic tissues, as detailed in Table S3. Additionally, colocalization analysis was conducted to exclude the possibility of confounding due to pleiotropy (PPH4 > 0.80) (Figure 2).

In the SMR results with instrument variants derived from blood, we observed that per 1-SD increase in TP53INP1 expression, there was a 17% increase in the risk of T2D (OR 1.17, 95% CI 1.11–1.23; PPH4 = 0.95), a 45% increased risk of DN (OR 1.45, 95% CI 1.22–1.73; PPH4 = 0.90), and a 37% increased risk of DR (OR 1.37, 95% CI 1.22–1.53; PPH4 = 0.82). Conversely, genetically predicted higher levels of expression of CYP2E1 (OR 0.77, 95% CI 0.68–0.86; PPH4 = 0.96) and G3BP2 (OR 0.94, 95% CI 0.91–0.97; PPH4 = 0.91) were inversely associated with T2D risk. Similarly, an increase of 1-SD in blood PRKD2 expression was connected with 29% (OR 0.71, 95% CI 0.62–0.81; PPH4 = 0.95) and 17% (OR 0.83, 95% CI 0.76–0.90; PPH4 = 0.97) diminished risk of DN and DR. In blood SUOX, MSRB3 and PRKAB1 expression per 1-SD increase was related to decreased risk of DN, with ORs (95% CIs) and respective PPH4 of 0.77 (95% CI 0.71–0.84; PPH4 = 0.99), 0.32 (95% CI 0.17–0.61; PPH4 = 0.93) and 0.89 (95% CI 0.83–0.94; PPH4 = 0.89); however, the higher levels of NOL3 (OR 2.04, 95% CI 1.38–3.02; PPH4 = 0.88) and CHEK1 (OR 2.21, 95% CI 1.42–3.44; PPH4 = 0.81) were genetically predicted positive association with DR risk.

Results for causal associations of OS-related genes with diabetes and complications in pancreas tissues are also presented in Figure 2. Genetically inferred expression of PTEN was inversely relevant to T1D risk in the pancreas (OR = 0.70, 95% CI 0.62–0.80; P_SMR_ 5.54 × 10^−8^). Moreover, we found that enhanced PRKD2 expression in pancreas tissues was connected to reduced DN risk (OR 0.67, 95% CI 0.55–0.81; P_SMR_ 4.05 × 10^−5^), and the increased expression of SUOX might lower the risk of DR (OR 0.87, 95% CI 0.83–0.92; P_SMR_ 1.10 × 10^−7^), which were consistent with the results in blood samples.

### 3.2. SMR Analysis of Cis-mQTL and Diabetes Outcomes

With adjustment for multiple testing and removal of associations with P-HEIDI < 0.01, we identified a total of 149 association signals at 68 unique genetic loci for T2D, 22 association signals at 14 unique loci for T1D, 45 association signals at 19 loci for DN, and 45 association signals at 22 loci for DR (Table S4). Among the 149 signals identified for T2D, strong colocalization evidence (PPH4 > 0.80) was observed for 29 signals near 16 unique genes. For T1D, colocalization analysis revealed that 5 CpG sites across 4 unique genes showed significant associations (Table 1). Notably, the effect estimates for diverse CpG sites within the same gene were not always consistent. For instance, per 1-SD increase in genetically predicted methylation of DUSP1 at cg17560677 was correlated with an increased risk of T1D (OR 1.52, 95% CI 1.18–1.94; PPH4 = 0.95), whereas an increase at cg22118147 was linked to a reduced risk of T1D (OR 0.76, 95% CI 0.64–0.89; PPH4 = 0.84).

**Figure 3:**
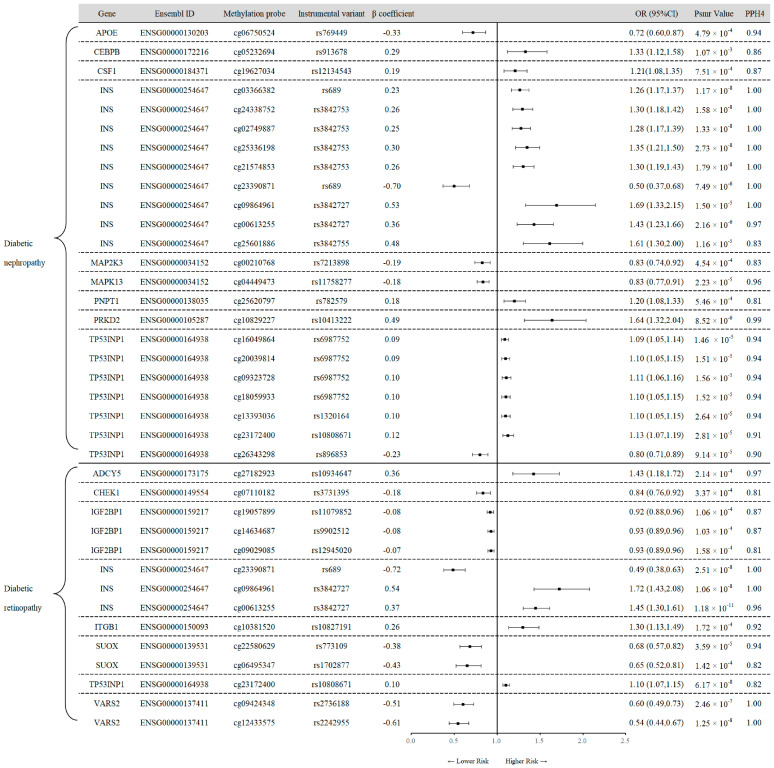
Mendelian randomization results for the association between the expression of OS-related gene methylations and the risk of diabetic nephropathy and diabetic retinopathy. OR, odds ratio; CI, confidence interval; PPH4, posterior probability of H4; APOE−apolipoprotein E; CEBPB−CCAAT enhancer-binding protein beta; CSF1−colony-stimulating factor 1; INS−insulin; MAP2K3−mitogen-activated protein kinase 3; MAPK13−mitogen-activated protein kinase 13; PNPT1−polyribonucleotide nucleotidyltransferase 1; PRKD2−protein kinase D2; TP53INP1−tumor protein P53-inducible nuclear protein 1; ADCY5−adenylate cyclase 5; CHEK1−checkpoint kinase 1; IGF2BP1−insulin-like growth factor 2 MRNA-binding protein 1; ITGB1−integrin subunit beta 1; SUOX−sulfite oxidase; VARS2−valyl-TRNA synthetase 2, mitochondrial.

Results for DN and DR risk associated with the OS gene on methylation level are shown in Figure 3. After Bayesian colocalization analysis (PPH4 > 0.80), 23 sites with 9 unique genes for DN were screened out (Figure 3). Our findings show a significant positive correlation between the methylation level of the gene CEBPB and the risk of DN (OR 1.33, 95% CI 1.12–1.58; PPH4 = 0.86). For diabetic retinopathy, there were 14 near-unique genes with high support evidence of colocalization, including ADCY5 (cg27182923), CHEK1 (cg07110182), IGF2BP1 (cg19057899, cg14634687, cg09029085), INS (cg23390871, cg09864961, cg00613255), ITGB1 (cg10381520), SUOX (cg22580629, cg06495347), TP53INP1 (cg23172400), and VARS2 (cg09424348, cg12433575). Meanwhile, the effect of instrumental variants of IGF2BP1, SUOX, and VARS2 on methylation levels was synergistic.

### 3.3. SMR Analysis of Cis-pQTL and Diabetes Outcomes

There were 11, 1, and 5 OS-related proteins separately associated with T2D, DN, and DR risk at P-FDR < 0.05 and P-HEIDI > 0.01 (Table S5). A genetically predicted higher level of ICAM1 (OR 1.05, 95% CI 1.03–1.08) was associated with an increased risk of DR, and a higher level of ARG1 was associated with a reduced risk of T2D, with an OR and 95% CI of 0.67 (0.57–0.78). Evidence of colocalization between ICAM1 and DR was observed (PPH4 = 0.91) (Table S5).

### 3.4. SMR Analysis for mQTL and eQTL Data

It is established that gene methylation affects gene expression. Here, we also conducted SMR analysis to explore the causal association between OS-related gene methylation and gene expression. This analysis involved mapping gene methylation to expression via shared genetic variants, providing insights into the regulatory mechanisms affecting gene functionality. After filtering the results with a significance threshold of P-FDR < 0.05 and P-HEIDI > 0.01, we established the associations between five DNA methylation CpG sites and the expression of three OS-related genes (Table S6) among the putative causal genes previously identified. The lower expression of TP53INP1 was associated with methylation of cg26343298, consistent with the protective role of cg26343298 methylation on type 2 diabetes and diabetic nephropathy. Similarly, an inverse association was observed with respect to gene methylation and expression in CHEK1 (cg07110182), confirming the negative effect of CHEK1 expression in diabetic retinopathy (OR 2.21, 95%CI 1.42–3.44). In addition, cg22580629 regulation of higher SUOX expression was associated with a lower risk of diabetic retinopathy (Table 2).

### 3.5. Sensitivity Analysis

The sensitivity analysis executed with “TwosampleMR” revealed that the F-statistics of the SNPs were all greater than 10, ranging from 29.7 to 3183.3 (Table S7). Further analysis confirmed the significant associations identified between the expression of CYP2E1, TP53INP1, and G3BP2 with T2D; PTEN with T1D; PRKD2 and TP53INP1 with DN; and SUOX, PRKD2, MSRB3, PRKAB1, NOL3, TP53INP1, and CHEK1 with DR (Table S8). The main analysis demonstrated consistent effect directions with our SMR results. Heterogeneity was observed for TP53INP1 with T2D, as well as SUOX, PRKD2, and PRKAB1 with DR. All genes showed no evidence of horizontal pleiotropy.

## 4. Discussion

In this study, we conducted Mendelian randomization (MR) and colocalization analyses to genetically assess the association of levels of methylation, expression, and protein abundance of oxidative stress-related genes with diabetes and its microvascular complications. At the genetic level, summary-data-based Mendelian randomization (SMR) and colocalization analyses identified potential causal relationships between oxidative stress genes and diabetes and microvascular complications. Further integration of DNA methylation and gene expression data highlighted three genes (TP53INP1, CHEK1, and SUOX) with plausible regulatory mechanisms. Our study provides evidence for potential mechanisms of oxidative stress gene loci, methylation, and expression in diabetes and related microvascular complications.

Diabetes can affect both the microvasculature and large blood vessels. Diabetic retinopathy and diabetic nephropathy are common microvascular complications, while macrovascular complications include atherosclerosis and coronary heart disease. Recent research increasingly supports the role of oxidative stress in the pathogenesis and progression of diabetes [27]. Oxidative stress is an important pathophysiological basis for the development of diabetes and its vascular complications [28], and possible mechanisms include increased ROS generation, endothelial dysfunction, release of inflammatory mediators, vascular smooth muscle cell proliferation and migration, cell apoptosis, and fibrosis [29,30,31]. Mechanistic studies of oxidative stress-related molecular pathways reveal major oxidative processes in diabetes, and pro-oxidation molecular cascade events involved in the activation of the advanced glycation end-product pathway and protein kinase C pathway are commonly upregulated in diabetes [32]. In addition, antioxidant stress is also important in diabetes and its vascular complications [33]. Antioxidant enzymes, such as superoxide dismutase (SOD), catalase (CAT), and glutathione peroxidase (GPx), are important defense mechanisms of the body against ROS. Clinically, oxidative stress-related biomarkers based on blood and tissues are widely used to diagnose, monitor, and predict diabetes activity [34].

In this study, we integrated mQTL and eQTL data to identify potential mediation mechanisms in which a SNP exerts an effect on the trait by altering the DNAm level, which then regulates the expression levels of a functional gene. We speculate that TP53INP1, CHEK1, and SUOX have reasonable regulatory mechanisms. TP53INP1 is a stress-induced protein that serves as a dual positive regulator of both transcription and autophagy. TP53INP1 can be induced by inflammation and stress and is overexpressed in a prediabetic rat model [35]. TP53INP1 is critically involved in cellular processes, including apoptosis and cell cycle regulation [36,37]. TP53INP1 and p53 may form a positive feedback loop under oxidative stress, while overexpression of p53 impairs insulin sensitivity, which is critically involved in the development of diabetes [38]. This may help us understand the mechanisms behind the association of TP53INP1 with a higher risk of diabetes from the perspective of glucose metabolism and insulin resistance. TP53INP1 has been identified as a potential susceptibility gene for diabetes based on previous GWAS [39,40]. Consistently, our findings confirmed that an increased TP53INP1 transcript level may contribute to the increased risk of type 2 diabetes (betaSMR = 0.15). In addition, epigenetic analysis has predicted a causal association between DNA methylation and type 2 diabetes [41,42]. Notably, our results suggest that cg26343298 methylation is protective against type 2 diabetes and diabetic nephropathy, and the cg26343298 methylation site negatively regulates TP53INP1 expression, indicating a correlation between DNA methylation, TP53INP1 expression, and diabetes risk.

Significantly, two other candidate genes, SUOX and CHEK1, which had not been intensively studied, were identified with a putative causal relationship with diabetic retinopathy. Sulfite oxidase, encoded by the SUOX gene, is an enzyme localized within mitochondria, dependent on molybdenum cofactor and heme, that catalyzes the essential oxidation of harmful sulfite to sulfate [43]. Mutation or defect of SUOX can lead to a decrease or loss of sulfite oxidase activity, resulting in symptoms like nervous system damage, eye abnormalities, birth defects, and heart diseases [43,44]. SUOX is a promising prognostic biomarker in a variety of cancers, such as hepatocellular carcinoma (HCC), intrahepatic cholangiocarcinoma (iCCA), etc. [45,46,47]. Genetically, SUOX may be a causal risk locus for Polycystic Ovary Syndrome (PCOS) [48], suggesting the potential of SUOX in endocrine disorder diseases. Moreover, the genetic variation of SUOX is one of the four T1D risk loci with genome-wide significance in the Chinese population [49]. Our results show that SUOX is genetically associated with diabetic retinopathy, and increased SUOX expression due to cg22580629 is associated with a decreased risk of diabetic retinopathy. in vitro and in vivo studies of severe sulfite oxidase deficiency indicate that the defective maturation of sulfite oxidase in mitochondria, likely to cause the impairment of its catalytic cysteine oxidation and transport in the vasculature, may result in toxic damage [50]. Homocysteine levels are higher in diabetic patients, which may exacerbate mitochondrial dysfunction and increase the development of diabetic retinopathy in a high-glucose environment [51]. These results highlight the importance of maintaining a balance of sulfide and cysteine to prevent or delay vision loss in patients with diabetes. Since cysteine catabolism is a major source of sulfite in humans, diabetic patients with abnormal SUOX genes may benefit from dietary restriction.

CHEK1 is a gene that encodes the human cell-cycle checkpoint kinase 1. When cells are subjected to DNA damage or other stresses, CHEK1 is activated and triggers a series of cellular stress responses, including suspending the cell cycle, repairing DNA damage, or inducing apoptosis [52]. Thus far, the research on CHEK1 has mainly focused on cancer, and CHEK1 inhibitors have unique potential in tumor-targeted therapy [53,54]. Results of our MR analysis indicated that the increase in cg07110182 methylation was associated with a reduced risk of DR by downregulating CHEK1 expression levels. Although direct evidence on CHEK1 and DR risk is unreported, DNA damage repair mechanisms have been implicated in diabetes and its complications [55]. Oxidative stress and inflammatory states can lead to damage to cellular DNA, which is more common in diabetes. Single-cell transcriptome studies in a mouse partial pancreatectomy model have observed high expression of DNA damage response factors, including CHEK1, during the β cell replication transition [56]. This persistent DNA damage can disrupt pancreatic cell function and consequently alter insulin secretion. Moreover, in diabetic vascular disease, endothelial cell impairment is significantly driven by oxidative stress and DNA damage resulting from hyperglycemia [57]. An endothelial cell model study assessed the cell cycle progression by demethylase and found that the G2/M phase of cell cycle progression by checkpoint kinase 1 (Chk1) phosphorylation was inhibited, which accompanied the activation of ATR/ATRIP signaling by H2AXS139 phosphorylation [58]. DNA damage mediated by overexpression of the oxidative stress gene CHEK1 may be involved in diabetic microvascular complications by injuring vascular endothelial cells.

Proteome-wide MR analysis predicts that ICAM1 may be a unique blood protein causally related to DR, revealing potential drug targets for DR. Intercellular adhesion molecule-1 (ICAM1), a membrane protein on immune and endothelial cells, can bind to lymphocyte function-associated antigen 1 (LFA-1) on leukocytes, thereby promoting the adhesion between leukocytes and endothelial cells and participating in intercellular adhesion and immune response. ICAM1 plays an important role in autoimmunity and islet rejection, leading to the death and dysfunction of islet β cells and causing the onset of diabetes [59]. Endothelial damage initiates both macroscopic and microscopic vascular dysfunction in diabetic patients, leading to prevalent vascular complications such as atherosclerosis, nephropathy, retinopathy, and neuropathy. Follow-up cohort studies have found that baseline levels of cell adhesion molecules are significantly associated with microvascular complications of T2D, which can be used as an indicator of microvascular complications in T2D patients [60]. Animal studies showed that diabetes significantly increased ICAM1 expression on the luminal surface of the vascular endothelium, with the highest induction in the mouse retina [61]. Studies of oxygen-induced retinopathy suggest that the early elevation of neutrophils induces an inflammatory and angiogenic response in the retina, which promotes severe damage to the retinal vasculature [62]. Immunological mechanism studies show that the up-regulation of ICAM1 and other inflammatory factors in the retina of diabetic retinopathy patients may be related to the pathway mediated by CD40 and its ligands [63,64,65]. Our analysis of oxidative stress-related proteomic data provides insights into the genetic pathophysiology of ICAM1 involvement in DR.

Considering that the pathogenesis of oxidative stress and diabetes is complex and multifaceted, the role of environmental factors in the disease cannot be ignored. There has been increasing concern in recent years about the question of the contribution of genes and the environment and their interactions in the development of diabetes [66]. This interaction underscores the complex interplay between genetic makeup and environmental conditions in determining health outcomes and disease susceptibility. Diet strategies and physical exercise [29] have been considered able to adjust the oxidative stress to prevent diabetes and its complications. The Mediterranean diet (MD) benefits from the presence of natural antioxidants to combat oxidative stress [67]. Physical exercise may regulate oxidation and antioxidant balance through ROS produced by skeletal muscle and vascular endothelial cells [68]. In the future, there may be hope for more scientific and personalized health guidance based on the results of genetic studies.

Overall, our MR study found several potential causal associations between methylation, expression, and protein abundance of OS-related genes and diabetes, as well as its comorbidities. The results highlight the pivotal roles of several genes in the pathogenesis of diabetes and its related disorders, revealing potential targets for pharmacological intervention. A strength of our research is the use of SMR and colocalization analysis, incorporating genetic variations to assess the causal effects of genes related to OS on methylation, expression, and protein levels. Consistent results from sensitivity analyses provide additional support for our findings. Moroever, it is crucial to recognize the constraints of our study. Because of the limited number of OS-related proteins, this research did not sufficiently explore the potential causal relationship between proteins involved in oxidative stress and the risks of DM and DN. Due to data limitations, we cannot calculate genetic risk scores (GRSs) based on individual genetic data. Additionally, information on genetic variants located on the X and Y chromosomes is not included in the eQTL and mQTL datasets. Finally, functional experiments are still required to validate our findings.

## 5. Conclusions

In summary, this SMR analysis explored the potential causal effects of OS-related genes on diabetes and its microvascular complications, highlighting the significant roles of TP53INP1, CHEK1, SUOX, and ICAM1 in the pathogenesis. The identified putative genes hold promise as potential pharmacological targets for diabetes; further research could explore details of the underlying biological mechanisms.

## Figures and Tables

**Table 1:** Mendelian randomization results for the association between the expression of OS-related gene methylations and diabetes mellitus risk.

Disease	Gene	Ensembl ID	Probe	β coefficient	OR (95%CI)	Psmr Value	PPH4
T2D	ADCY5	ENSG00000173175	cg27182923	0.29	1.34 (1.19, 1.50)	6.66 × 10^−7^	0.97
	APAF1	ENSG00000120868	cg10957001	−0.02	0.98 (0.97, 0.99)	6.08 × 10^−6^	0.89
	BCL2L11	ENSG00000153094	cg04202892	−0.04	0.96 (0.95, 0.98)	1.06 × 10^−5^	0.93
	BCL2L11	ENSG00000153094	cg04780086	−0.14	0.87 (0.81, 0.94)	2.09 × 10^−4^	0.92
	BCL2L11	ENSG00000153094	cg18646521	−0.08	0.93 (0.89, 0.96)	4.35 × 10^−5^	0.92
	BCL2L11	ENSG00000153094	cg00997280	0.09	1.09 (1.04, 1.15)	1.08 × 10^−3^	0.87
	BDNF	ENSG00000176697	cg00298481	0.15	1.16 (1.06, 1.28)	9.61 × 10^−4^	0.84
	CAMK2G	ENSG00000148660	cg07512258	0.11	1.12 (1.06, 1.18)	5.88 × 10^−5^	0.90
	CDKN1A	ENSG00000124762	cg24425727	0.03	1.03 (1.02, 1.05)	8.74 × 10^−5^	0.86
	CDKN1A	ENSG00000124762	cg11920449	0.03	1.03 (1.02, 1.05)	8.64 × 10^−5^	0.86
	CDKN1A	ENSG00000124762	cg03714916	0.10	1.10 (1.04, 1.16)	3.00 × 10^−4^	0.86
	CYB5R3	ENSG00000100243	cg08690876	−0.05	0.95 (0.93, 0.97)	1.16 × 10^−5^	0.86
	CYB5R3	ENSG00000100243	cg25044876	−0.12	0.89 (0.83, 0.95)	2.38 × 10^−4^	0.80
	CYP2E1	ENSG00000130649	cg01465364	−0.15	0.86 (0.79, 0.93)	2.40 × 10^−4^	0.97
	CYP2E1	ENSG00000130649	cg19571004	−0.14	0.87 (0.81, 0.94)	1.35 × 10^−4^	0.97
	HSF1	ENSG00000185122	cg18814314	−0.04	0.97 (0.95, 0.98)	2.16 × 10^−6^	0.90
	IGF2BP1	ENSG00000159217	cg09029085	−0.05	0.95 (0.93, 0.97)	7.65 × 10^−8^	0.98
	IGF2BP1	ENSG00000159217	cg14634687	−0.05	0.95 (0.93, 0.97)	1.04 × 10^−7^	0.97
	IGF2BP1	ENSG00000159217	cg19057899	−0.05	0.95 (0.93, 0.97)	1.27 × 10^−7^	0.97
	INSR	ENSG00000171105	cg10381200	0.20	1.22 (1.11, 1.33)	1.76 × 10^−5^	0.91
	MAP2K3	ENSG00000034152	cg00210768	−0.06	0.94 (0.91, 0.97)	1.55 × 10^−4^	0.85
	NUDT1	ENSG00000106268	cg12823233	−0.03	0.97 (0.96, 0.98)	3.47 × 10^−6^	0.83
	PPARG	ENSG00000132170	cg04632671	0.26	1.30 (1.19, 1.42)	8.63 × 10^−9^	0.98
	TP53INP1	ENSG00000164938	cg16049864	0.04	1.04 (1.02, 1.05)	5.47 × 10^−9^	0.98
	TP53INP1	ENSG00000164938	cg20039814	0.04	1.04 (1.03,1.05)	6.08 × 10^−9^	0.98
	TP53INP1	ENSG00000164938	cg13393036	0.04	1.04 (1.03, 1.06)	6.51 × 10^−9^	0.98
	TP53INP1	ENSG00000164938	cg26343298	−0.10	0.91 (0.88, 0.94)	1.88 × 10^−7^	0.95
	TP53INP1	ENSG00000164938	cg23172400	0.05	1.05 (1.03, 1.07)	2.13 × 10^−8^	0.95
	TSFM	ENSG00000123297	cg12113251	0.11	1.11 (1.04, 1.19)	1.73 × 10^−3^	0.84
T1D	BDNF	ENSG00000176697	cg10635145	−0.17	0.84 (0.77, 0.92)	3.16 × 10^−4^	0.81
	DUSP1	ENSG00000120129	cg17560677	0.42	1.52 (1.18, 1.94)	1.08 × 10^−3^	0.95
	DUSP1	ENSG00000120129	cg22118147	−0.28	0.76 (0.64, 0.89)	8.87 × 10^−4^	0.84
	SRXN1	ENSG00000172070	cg15557840	−0.06	0.94 (0.90, 0.97)	5.49 × 10^−4^	0.81
	SUOX	ENSG00000139531	cg22580629	−0.66	0.52 (0.38, 0.69)	1.22 × 10^−5^	0.93

Abbreviations: T2D, type 2 diabetes; T1D, type 1 diabetes; OR, odds ratio; CI, confidence interval; PPH4, posterior probability of H4; ADCY5−adenylate cyclase 5; APAF1−apoptotic peptidase-activating factor 1; BCL2L11−BCL2-like 11; BDNF−brain-derived neurotrophic factor; CAMK2G−calcium-/calmodulin-dependent protein kinase II gamma; CDKN1A−cyclin-dependent kinase inhibitor 1A; CYB5R3−cytochrome B5 reductase 3; CYP2E1−cytochrome P450 family 2 subfamily E member 1; HSF1−heat-shock transcription factor 1; IGF2BP1−insulin-like growth factor 2 MRNA-binding protein 1; INSR−insulin receptor; MAP2K3−mitogen-activated protein kinase 3; NUDT1−nudix hydrolase 1; PPARG−peroxisome proliferator-activated receptor gamma; TP53INP1−tumor protein P53-inducible nuclear protein 1; TSFM−translation elongation factor, mitochondrial; BDNF−brain-derived neurotrophic factor; DUSP1−dual specificity phosphatase 1; SRXN1−sulfiredoxin 1; SUOX−sulfite oxidase.

**Table 2:** Integrating evidence from multi-omics levels.

Outcome	Gene	eQTL → GWAS			Probe	mQTL → GWAS			mQTL → eQTL			
		OR (95% CI)	*p* Value	PPH4		OR (95% CI)	*p* Value	PPH4	Beta	se	*p* Value	PPH4
T2D	TP53INP1	1.17 (1.11, 1.23)	1.62 × 10^−8^	0.95	cg26343298	0.91 (0.88, 0.94)	1.88 × 10^−7^	0.95	−0.62	0.05	2.01 × 10^−31^	0.99
DN		1.45 (1.22, 1.73)	3.12 × 10^−5^	0.90		0.80 (0.71, 0.89)	9.14 × 10^−5^	0.90				
DR	SUOX	0.77 (0.71, 0.84)	4.57 × 10^−9^	0.99	cg22580629	0.68 (0.57, 0.82)	3.59 × 10^−5^	0.94	1.52	0.24	2.19 × 10^−10^	0.97
DR	CHEK1	2.21 (1.42, 3.44)	4.81 × 10^−4^	0.81	cg07110182	0.84 (0.76, 0.92)	3.37 × 10^−4^	0.81	−0.23	0.03	3.43 × 10^−12^	0.89

Abbreviations: QTL, quantitative trait loci; PPH4, posterior probability of H4; T2D, type 2 diabetes; DN, diabetic nephropathy; DR, diabetic retinopathy; TP53INP1−tumor protein P53-inducible nuclear protein 1; SUOX−sulfite oxidase; CHEK1−checkpoint kinase 1.

## Data Availability

The data used for Mendelian Randomization (MR) analysis can be obtained from the referenced peer-reviewed studies and are listed in Supplementary Table S1.

## Supplementary Materials

The following supporting information can be downloaded at: https://www.mdpi.com/article/10.3390/antiox13080903/s1. Table S1. Information on included studies and consortia. Table S2. 817 OS-related genes with a relevance score ≥7 obtained from GeneCards. Table S3. SMR and colocalization results of the association between expression of OS-related genes and diabetes mellitus and microvascular complications (FDR < 0.05 and P-HEIDI > 0.01). Table S4. SMR and colocalization results of the association between DNA methylation of OS-related genes and diabetes mellitus and microvascular complications (FDR < 0.05 and P-HEIDI > 0.01). Table S5. SMR and colocalization results of the association between protein expression of OS-related genes and diabetes mellitus and microvascular complications (FDR < 0.05 and P-HEIDI > 0.01). Table S6. SMR and colocalization results of the association between DNA methylation and gene expression of OS-related genes (FDR < 0.05 and P-HEIDI > 0.01). Table S7. SNPs used as instrumental variables for two-sample MR analysis and F-statistic. Table S8. Sensitivity analysis used two-sample MR analysis on the association between OS-related gene expression and diabetes mellitus and microvascular complications.
